# Characteristic patterns of emergency ambulance assignments for older adults compared with adults requiring emergency care at home in Sweden: a total population study

**DOI:** 10.1186/s12873-020-00387-y

**Published:** 2020-12-02

**Authors:** Anna Hjalmarsson, Mats Holmberg, Margareta Asp, Gunnel Östlund, Kent W. Nilsson, Birgitta Kerstis

**Affiliations:** 1 School of Health, Care and Social Welfare, Mälardalen University, Eskilstuna Västerås, Sweden; 2 Faculty of Health and Life Sciences, Linneaus University, Växjö, Sweden; 3 Centre for Clinical Research Sörmland, Uppsala University, Eskilstuna, Sweden; 4 Department of Ambulance Service, Region Sörmland, Eskilstuna, Sweden; 5 Centre for Clinical Research Västerås, Uppsala University, Västmanland County Hospital, Västerås, Sweden

**Keywords:** Ambulance, Characteristics, Emergency medical services, Older adults

## Abstract

**Background:**

Since the vast majority of older adults in Sweden live in their private homes throughout life, the emergency medical services need to adapt accordingly. Hence, we aimed to describe characteristic patterns of dyadic staffed emergency ambulance assignments for older adults aged > 70 years compared with adults aged 18–69 years requiring emergency care at home in Sweden.

**Methods:**

A descriptive retrospective study was performed using anonymized registry data from the emergency medical services in a region of Sweden during 2017–2018. One-sample χ^2^ test, one-way analysis of variance, and binary logistic regression models were used for investigating group differences. Variables for analysis were age, gender, clinical assessments, on-scene time, priority levels, result of response, and temporal patterns.

**Results:**

Of all included emergency ambulance assignments (*n* = 28,533), 59.9% involved older adults, of which 53.8% were women. The probability for older adults to receive the highest priority was decreased for both dispatch (*p* < 0.001, odds ratio [OR] 0.63, 95% confidence interval [CI] 0.59–0.66), and transport priorities (*p* < 0.001, OR 0.74, 95% CI 0.68–0.80). Older adults were more likely to receive dispatch priority levels 2 (*p* < 0.001, OR 1.48, 95% CI 1.40–1.56), and 3 (*p* < 0.001, OR 1.73, 95% CI 1.46–2.06). The older adults were similarly more likely to receive transport priority level 3 (*p* < 0.001, OR 1.40, 95% CI 1.28–1.52) compared with adults. Age had a small but additive effect in relation to on-scene time (*p* < 0.001, R^2^ = 0.01, F = 53.82). Distinguishing initial clinical assessments for older adults were circulatory, respiratory, trauma, infection, and nonspecific assessments. Emergency ambulance assignments for older adults were more frequently occurring on Mondays (*p* < 0.001, χ^2^ = 232.56), and in the 08:00–11:59 interval (*p* < 0.001, χ^2^ = 1224.08).

**Conclusion:**

The issues of the lower priority level preponderance, and the decreased probability for receiving the highest priority warrant further attention in future research and clinical practice.

## Background

There is an ongoing shift in population demographics worldwide, from younger to older age [1]. Therefore, the World Health Organization strongly recommends that health care systems globally redesign their services to better fulfill older people’s needs and to promote their independence [1]. Older age entails prevalence of cognitive and physical impairments, chronic medical conditions, and frailty, thus increasing the need for emergency ambulance care [2–6]. Currently, approximately 1.5 million people in Sweden are aged > 70 years [7], a population that is estimated to rise by 25% over the next 15 years [8]. In addition, the population of the current 0.5 million people aged > 80 years [7] is estimated to rise by 61% in the same period [8]. Life expectancy in Sweden is high compared with many other countries. A global ranking places Sweden 11th [9] with an average life expectancy of 84.7 years for women and 81.3 years for men [10]. By 2060, the life expectancies of Swedish women and men are estimated to reach 89.1 and 86.7 years respectively [11]. Due to targeted political governance, the vast majority of older adults live in their own private homes throughout life, only about 80,000 older adults live in nursing homes [12]. Hence, the emergency needs of the older adult population living at home will substantially impact future ambulance care.

The Swedish emergency medical services (EMS) providing emergency ambulance care are regulated by law, which states that high-quality care is to be provided to all, assuring safety, equality, and patient participation [13, 14]. Swedish EMS personnel are the frontline providers of this stated care and include primarily registered nurses with a bachelor’s degree and specialist nurses with postgraduate education within, for example, prehospital, anesthetic, or intensive care (henceforth, registered and specialist nurses are referred to as RNs). The Swedish EMS RNs have an autonomous prehospital medical and caring responsibility, requiring specialist competence [15]. The context of prehospital emergency care is complex, and the RNs need to adjust to organizational requirements of rapid responses and available care options in relation to the patient’s medical or personal needs, and safety [15]. The prehospital assessment is based on very limited background information, and entails considerations of risks versus benefits influenced by the EMS RN’s competence, experience, organizational support, and guidelines [16, 17]. The EMS RNs carry out an initial clinical assessment of the patient’s symptoms and emergency need on scene, to determine the adequate level of emergency care and transport priority if conveyance to hospital is deemed necessary. The options for level of care are limited in Sweden, and generally include conveyance to hospital or leaving the older adult at home. Older adults are challenging to clinically assess, often displaying low acuity conditions [18, 19] or atypical or masked symptoms due to conditions such as polypharmacy, dementia, or chronic diseases [20]. The prehospital assessment and the level of care provided, plays a paramount role for the health outcome of older adults.

Previous research focusing on older adults receiving ambulance care reported such assignments to be prolonged compared with those for younger adults [21], and that conveyance rates were high [5, 21]. An Australian study reporting temporal patterns of ambulance demand for community dwelling older adults, found that such calls peaked on Mondays around 11:00 and were more frequent in the Australian winter and spring [5]. A Swedish study of EMS assignments for older adults found that such adults were more frequently assessed as having nonspecific conditions, and that age and gender influenced the assessments [22].

Swedish EMS research targeting specific emergency needs of the increasing population of older adults living at home is however scarce. This study provides an understanding of prevalent emergency ambulance assignments for older adults living at home in Sweden. Since the Swedish EMS is a statutory welfare service, defined to be safe, equal, and of high quality for all, it is of likewise importance to describe potential inequalities between populations. Such knowledge would further enable interventions beneficial for older adults, in accordance with the legislative liability of the EMS. To identify characteristics representative of assignments for older adults, we performed a comparative analysis between older adults and the general adult population. Hence, the overall study aim was to describe characteristic patterns of dyadic staffed emergency ambulance assignments for older adults aged > 70 years compared with adults aged 18–69 years requiring emergency care at home. Age group differences in the following were investigated: age and gender frequency; result of response; priority levels; clinical assessments; on-scene time; and temporal patterns.

## Methods

### Study design

The present study had a descriptive 2-year range retrospective design. Registry data from the Swedish EMS in the region of Sörmland was analyzed, comprising all EMS assignments from January 2017 to December 2018. Ethical approval was obtained by the Swedish Ethical Review Authority (Dnr: 2019–02027), and access permission to anonymized registry data was provided by the manager of the EMS in the region.

### Study setting

The region included in this study is one of 21 regions in Sweden. This medium-sized region (ca. 6000 km^2^) is situated southwest of Stockholm, and consists of nine municipalities covering rural and urban areas. The region has a population of approximately 300,000 inhabitants, with 85% of them living in urban areas [23]. Approximately 17% of the inhabitants are older adults aged > 70 years, of which 54% are women [24]. The EMS of the region cover six ambulance stations, each located in one of the six largest municipalities. On weekdays, the EMS have at their disposal 16 daytime (08:00–18:00) emergency ambulance vehicle units and 11 nighttime (18:00–08:00) units. At weekends, 13 and 10 vehicles are available, respectively. Swedish EMS personnel mainly work in dyads, and besides RNs, emergency medical technicians (EMTs) mainly trained as assistant nurses are a common part of the team [25]. All dyadic staffed emergency ambulances in Sweden are considered advanced life support (ALS) units [15], and the RNs are licensed for drug administration [25]. Single responder ambulances have been implemented in the Swedish EMS, managed by one RN alone and not used for conveyance [26]. The Swedish EMS utilize the Rapid Emergency Triage and Treatment System (RETTS©) as an instrument for triage [27]. EMS physicians mainly serve as medical advisors, and are not part of the ambulance team [25].

Three descending levels of emergency are used for prioritizing EMS assignments by the emergency medical communication centers (EMCC) receiving medical emergency calls: 1) life-threatening or possibly life-threatening; 2) not life-threatening, requiring advanced care and conveyance; and 3) not life-threatening, advanced care and conveyance required within a reasonable time [28]. Level 1 is a high priority level indicating unstable vital signs, and levels 2 and 3 are considered lower priority levels indicating stable vital signs.

Swedish health care is part of a welfare system financed by the regions through income taxes and government support. The cost for EMS responses is settled by the regions. In the region of the present study, ambulance responses are free of charge for citizens aged 0–19 and > 85 years and subsidized for other citizens (ca. €37/US$41 per response).

### Study population

**Fig. 1:**
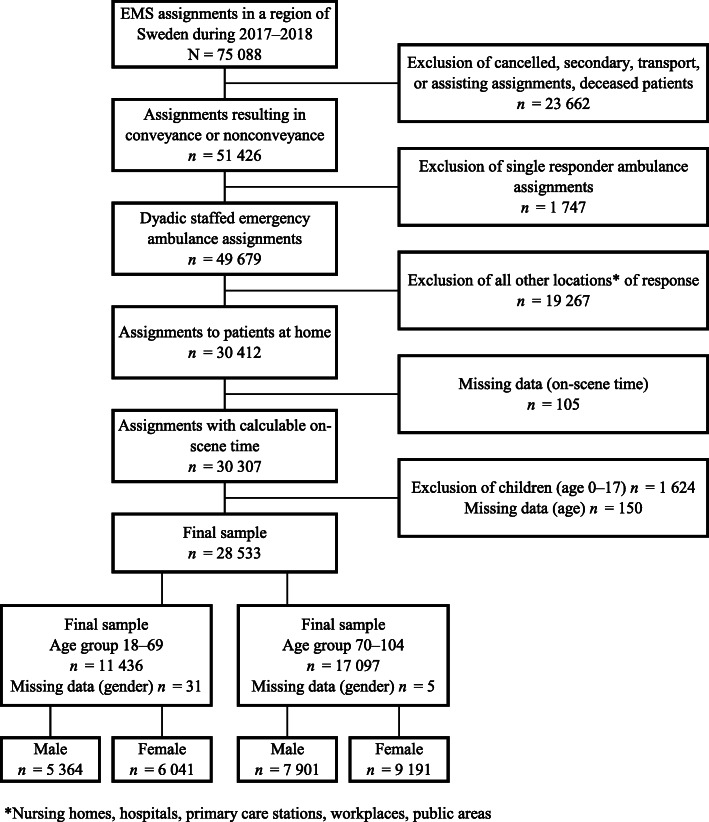
Flow chart of final sample. The sample (*n* = 28,533) represents primary dyadic staffed emergency ambulance assignments for adults and older adults requiring emergency care at home, resulting in conveyance or nonconveyance

The data comprise all assignments performed by the EMS (*N* = 75,088) in 2017–2018. The final sample (*n* = 28,533) for analysis represents primary dyadic staffed ALS emergency ambulance assignments for adults requiring emergency care at home, resulting in conveyance or nonconveyance, with entries made in clinical records for possible calculation of on-scene time (Fig. 1). In the present study, older adults are defined as those aged > 70 years, and adults as aged 18–69 years. This age cutoff has been previously used in similar research [29].

### Variables for analysis

A total of seven assignment variables were processed for analysis: age, gender, result of response, priority levels, on-scene time, clinical assessments, and temporal patterns. Age was analyzed as a continuous variable or stratified into two age groups: adults (18–69 years) and older adults (70–104 years). Age was additionally stratified into eight age groups for one-way analysis of variance, and for visual display of responses in relation to age (18–29, 30–39, 40–49, 50–59, 60–69, 70–79, 80–89, and 90–104 years). The dichotomous age group variable is used as the main predictor in the logistic regression models.

Result of response is denoted as conveyance (transport to hospital) or nonconveyance (remain at home). The variable is used as outcome, and as predictor in other logistic regression models. Dispatch priority denotes the priority level assigned to the EMS response by the EMCC receiving the medical emergency call. Transport priority denotes the priority level assigned to the patient by the EMS RNs when conveyed to hospital. The three priority levels were dichotomized for binary logistic regression modeling: level 1/level 2 or 3, level 2/level 1 or 3, and level 3/level 1 or 2. The level 1/level 2 or 3 variable is used as predictor in other logistic regression models.

The clinical assessments in the registry data encompassed 134 assessments entered by the EMS RNs. These assessments were clustered into 14 categories in accordance with the emergency signs and symptoms (ESS) grouping used by the EMS in the region [27]. Of the 14 categories, two were without any clinical assessment (transfer, other) and therefore presented as missing cases. For analysis using binary logistic regression, the assessments were dichotomized (assessment/all other assessments). The variable is used as outcome, and as predictor in other logistic regression models.

Ambulance on-scene time denotes the time in minutes spent in the home of the adults and older adults or in the ambulance on the scene, ranging from 1 to 235 min. The variable was calculated by subtracting ambulance arrival time from departure time, in consensus with the registry manager of the EMS. The variable is used for one-way analysis of variance and as predictor in the logistic regression models.

Temporal patterns were categorized into three time periods: diurnal, weekly, and seasonal. The diurnal variable was further divided into two or six periods of time: 08:00–17:59 (daytime) and 18:00–07:59 (nighttime) in accordance with the EMS ambulance disposition, and in 4-h intervals: 00:00–03:59, 04:00–07:59, 08:00–11:59, 12:00–15:59, 16:00–19:59, and 20:00–23:59. The weekly variables were stratified into two or seven time periods: weekdays (Monday–Friday) and weekend (Saturday–Sunday), and each day of the week. The seasonal variable was stratified into four categories: winter (December–February), spring (March–May), summer (June–August), and autumn (September–November). The variable is used in one-sample χ^2^ test, and the dichotomous daytime/nighttime variable is used as predictor in the logistic regression models, referred to as time of day.

### Statistical analysis

Descriptive statistics are presented using frequencies and percentages for categorical variables and using means and standard deviations for continuous variables. Nonparametric variable correlations were analyzed using one-sample χ^2^ test, and one-way analysis of variance was used for age and on-scene time correlations, with supplementary Scheffe’s post hoc for between group multiple comparison. Binary logistic regression models were used to analyze the dichotomized outcome variables priority level, result of response, and initial clinical assessment. The chosen predictor variables were discussed among the researchers, and considered to be of importance for the outcome variables. A *p*-value < 0.05 was considered statistically significant. All analyses were performed using IBM SPSS Statistics (version 24.0; IBM SPSS, Armonk, NY, USA).

## Results

**Fig. 2:**
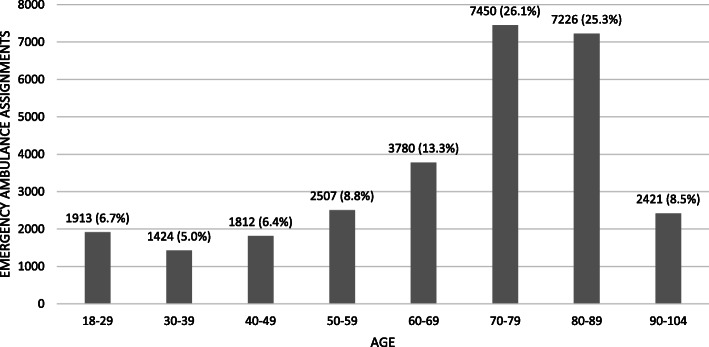
Dyadic staffed emergency ambulance assignments to private homes in the region of Sörmland in Sweden 2017–2018, stratified by age groups

Of the 28,533 included dyadic staffed emergency ambulance assignments to private homes in the region during 2017–2018, 17,097 (59.9%) involved older adults aged > 70 years (mean = 81.3, standard deviation = 7.0, range = 70–104 years), of which 53.8% were women. In relation to age, there was a considerable increase in assignments for older adults aged 70–79 and 80–89 years (Fig. 2).

Older adults were more likely to be conveyed to hospital compared to the adult population (*p* < .001, adjusted odds ratio [adjOR] 1.79, 95% confidence interval [CI] 1.33–2.40) (Table 2), the conveyance rate being 89.4% within the older adult age group compared with 82.6% in the adult age group (Table 1). In the adult population, 46.0% received the highest priority, in contrast to only 37.3% in the older adult group (Table 1). The likelihood for older adults to receive the highest priority level was reduced for both dispatch (*p* < .001, adjOR 0.63, 95% CI 0.59–0.66), and transport priorities (*p* < .001, adjOR 0.74, 95% CI 0.68–0.80) (Table 2). The probability for receiving a dispatch priority level 2 (*p* < .001, adjOR 1.48, 95% CI 1.40–1.56) or 3 (*p* < .001, adjOR 1.73, 95% CI 1.46–2.06) was increased for the older adults (Table 2), constituting 59.9 and 2.7% of the older adults compared with 52.1 and 1.9% in the adult population (Table 1). The older adult group were similarly more likely to receive a transport priority level 3 (*p* < .001, adjOR 1.40, 95% CI 1.28–1.52) compared with the adults (Table 2). The proportion of transport priority level 2 were similar in both age groups, 72.9% of the adults and 73.1% of the older adults received priority level 2, and there was no difference in odds ratio (Table 2).

**Table 1:** Characteristics of emergency ambulance assignments for adults and older adults at home in Sweden

Age group	Adults (18–69) *n* = 11,436	Older adults (70–104) *n* = 17,097
Age (years)		
Mean (SD)	48.9 (15.3)	81.3 (7.0)
Male (SD)	50.5 (14.9)	80.5 (6.6)
Female (SD)	47.5 (15.5)	81.9 (7.3)
Median	52	81
Male	54	80
Female	49	82
Gender *n* (% within group)^a^		
Male	5364 (47.0)	7901 (46.2)
Female	6041 (53.0)	9191 (53.8)
Result of response *n* (% within group)		
Conveyance	9443 (82.6)	15,279 (89.4)
Non-conveyance	1993 (17.4)	1818 (10.6)
Priority level *n* (% within group)^b^		
Dispatch priority level 1	5256 (46.0)	6382 (37.3)
Dispatch priority level 2	5961 (52.1)	10,247 (59.9)
Dispatch priority level 3	219 (1.9)	468 (2.7)
Transport priority level 1	1391 (14.6)	2045 (13.3)
Transport priority level 2	6956 (72.9)	11,239 (73.1)
Transport priority level 3	1199 (12.6)	2098 (13.6)

^a^Missing cases: gender; adults 31, older adults 5

^b^Patients receiving transport priority without recorded entries of conveyance; adults 103, older adults 103

Descriptive statistics presented for age, gender, result of response, and priority level variables. Percentage in brackets display within group proportion

**Table 2:** Priority levels and result of response for older adults at home in Sweden

Adults (18–69) *n* = 11,436 Older adults (70–104) *n* = 17,097				Unadjusted		Adjusted
Priority level	Age group	(% within group)	***p***	OR (95% CI)	***p***	OR (95% CI)
Dispatch Priority level 1	Adults	(46.0)		Ref		Ref
	Older adults	(37.3)	< .001	0.70 (0.67–0.74)	< .001	**0.63 (0.59–0.66)**
Dispatch priority level 2	Adults	(52.1)				
	Older adults	(59.9)	< .001	1.37 (1.31–1.44)	< .001	**1.48 (1.40–1.56)**
Dispatch priority level 3	Adults	(1.9)				
	Older adults	(2.7)	< .001	1.44 (1.23–1.70)	< .001	**1.73 (1.46–2.06)**
Transport priority level 1	Adults	(14.6)				
	Older adults	(13.3)	.005	0.90 (0.84–0.97)	< .001	**0.74 (0.68–0.80)**
Transport priority level 2	Adults	(72.9)				
	Older adults	(73.1)	.740	1.01 (0.95–1.07)	.820	0.99 (0.93–1.06)
Transport priority level 3	Adults	(12.6)				
	Older adults	(13.6)	.016	1.10 (1.02–1.19)	< .001	**1.40 (1.28–1.52)**
Result of response						
Conveyance	Adults	(82.6)				
	Older adults	(89.4)	< .001	1.77 (1.66–1.90)	< .001	**1.79 (1.33–2.40)**

Binary logistic regression models for priority level and result of response variables presented as unadjusted, and adjusted odds ratios (OR) with 95% confidence interval (CI). Independent variables adjusted for in the priority level models are gender, initial clinical assessment, on-scene time, result of response and time of day. Independent variables adjusted for in the result of response model are gender, initial clinical assessment, on-scene time, priority level and time of day. Percentage in brackets displays proportion within age group. Significant adjusted odds ratios differentiating the older adult group are highlighted in bold text

Circulatory, respiratory, trauma, infection, and nonspecific assessments were predominant in the older adult age group, and the probability for all assessments but circulatory and trauma were more than doubled (Table 3). Eye, ear, nose and throat assessments had a similar increased likelihood, but represented only 1.1% of the assessments within the age group.

**Table 3:** Initial clinical assessments for adults and older adults at home in a region of Sweden

Adults (18–69) *n* = 11,436 Older adults (70–104) *n* = 17,097				Unadjusted		Adjusted
Initial clinical assessment^a^	Age group	***n*** (%)	***p***	OR (95% CI)	***p***	OR (95% CI)
Circulatory (e.g. chest pain, cardiac arrest/dysrhythmia, hypertension)	Adults	1997 (17.5)	.240	Ref		Ref
	Older adults	2894 (16.9)		0.96 (0.91–1.03)	.023	**1.09 (1.01–1.17)**
Eye, ear, nose, throat (e.g. bleeding, pain, foreign body)	Adults	64 (0.6)				
	Older adults	190 (1.1)	< .001	2.00 (1.50–2.65)	< .001	**2.60 (1.86–3.65)**
Genital (gynecology, urology)	Adults	194 (1.7)				
	Older adults	292 (1.7)	.941	1.01 (0.84–1.21)	302	1.11 (0.91–1.36)
Infection	Adults	581 (5.1)				
	Older adults	1732 (10.1)	< .001	2.11 (1.91–2.32)	< .001	**2.05 (1.84–2.27)**
Medical (e.g. anemia, diabetic symptoms, Addison’s disease, immune deficiency)	Adults	1109 (9.7)				
	Older adults	1189 (7.0)	< .001	0.70 (0.64–0.76)	< .001	0.63 (0.57–0.70)
Neurology (e.g. stroke, headache, seizure, loss of consciousness, dizziness)	Adults	1952 (17.1)				
	Older adults	2727 (16.0)	.012	0.92 (0.87–0.98)	.120	0.95 (0.88–1.01)
Nonspecific	Adults	216 (1.9)				
	Older adults	673 (3.9)	< .001	2.13 (1.83–2.49)	< .001	**2.08 (1.73–2.50)**
Orthopedic (e.g. musculoskeletal pain)	Adults	585 (5.1)				
	Older adults	824 (4.8)	.255	0.94 (0.84–1.05)	< .001	0.71 (0.63–0.81)
Psychiatric (e.g. psychiatric disorders, suicidality, anxiety, drug abuse)	Adults	520 (4.5)				
	Older adults	194 (1.1)	< .001	0.24 (0.20–0.29)	< .001	0.21 (0.17–0.26)
Respiratory (e.g. respiratory distress, dyspnea, hyperventilation)	Adults	731 (6.4)				
	Older adults	2232 (13.1)	< .001	2.20 (2.02–2.40)	< .001	**2.41 (2.19–2.66)**
Surgical (e.g. gastrointestinal hemorrhage, abdominal pain, nausea/vomiting)	Adults	1740 (15.2)				
	Older adults	1654 (9.7)	< .001	0.60 (0.55–0.64)	< .001	0.58 (0.54–0.68)
Trauma (physical injury)	Adults	1075 (9.4)				
	Older adults	1998 (11.7)	< .001	1.28 (1.18–1.38)	.023	**1.10 (1.01–1.20)**

^a^Patients not receiving initial clinical assessment; adults 672, older adults 498

Binary logistic regression models for each assessment presented as unadjusted, and adjusted odds ratios (OR) with 95% confidence interval (CI). Independent variables adjusted for in the models are gender, on-scene time, priority level, result of response and time of day. Adjusted odds ratios characterizing initial clinical assessments for older adults are highlighted in bold text. The proportion of adults and older adults receiving the assessment within the age group is displayed as *n* (%)

**Fig. 3:**
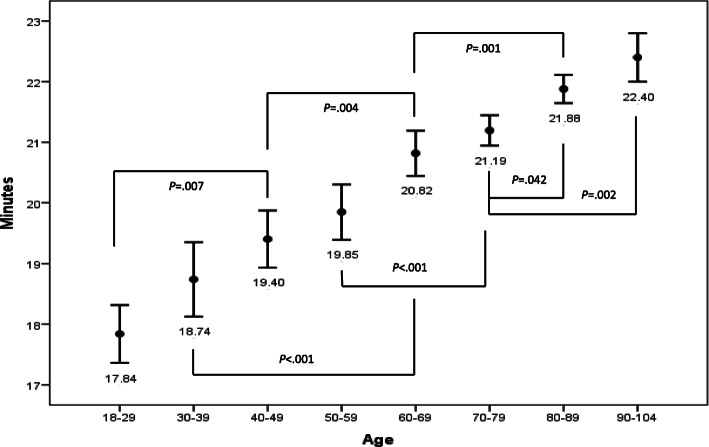
Mean and 95% confidence interval for emergency ambulance on-scene time using one-way analysis of variance, and Scheffe’s post hoc. Age groups having significant longer on-scene time are displayed with *p*-values in the figure

Figure 3 displays the impact of age on emergency ambulance on-scene time. Age had a small although additive effect in relation to on-scene time (*p* < 0.001, R^2^ = 0.01, F = 53.82). The *p*-values displayed in Fig. 3, indicate age groups with significantly longer on-scene time.

**Fig. 4:**
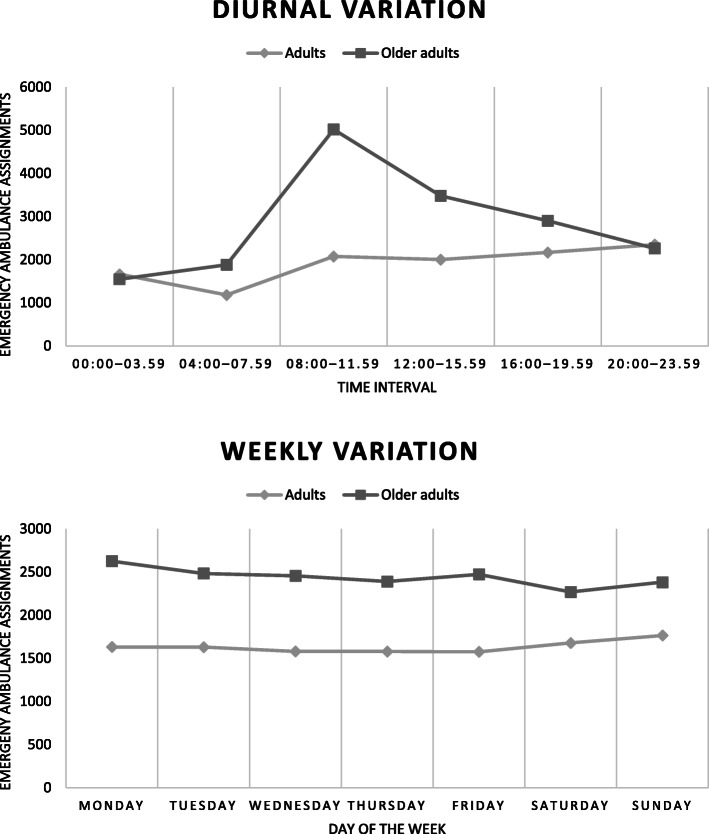
Temporal patterns of emergency ambulance assignments for adults and older adults at home, in the region of Sörmland in Sweden 2017–2018

Age group differences were found in all temporal variables (Table 4). Compared to the adult population, emergency ambulance assignments for older adults were more frequent during daytime, and in the 08:00–11.59 interval (Fig. 4). An older adult preponderance was additionally found on weekdays, on Mondays (Fig. 4), and in the winter.

**Table 4:** Temporal patterns of emergency ambulance assignments for adults and older adults at home in Sweden

Age group	Adults (18–69) *n* = 11,436	Older adults (70–104) *n* = 17,097	*p*	χ^2^
Diurnal patterns *n* (% within group)				
00:00–03.59	1661 (14.5)	1547 (9.0)	.044	4.05
04:00–07.59	1182 (10.3)	1882 (11.0)	< .001	159.92
08:00–11.59	2074 (18.1)	5021 (29.4)	< .001	**1224.08**
12:00–15.59	2002 (17.5)	3480 (20.4)	< .001	398.48
16:00–19.59	2167 (18.9)	2904 (17.0)	< .001	107.11
20:00–23.59	2350 (20.5)	2263 (13.2)	.200	1.64
08:00–17.59 (daytime)	5087 (44.5)	9887 (57.8)	< .001	**1538.67**
18:00–07.59 (nighttime)	6349 (55.5)	7210 (42.2)	< .001	54.67
Weekly patterns *n* (% within group)				
Monday	1631 (14.3)	2626 (15.4)	< .001	**232.56**
Tuesday	1629 (14.2)	2483 (14.5)	< .001	177.36
Wednesday	1580 (13.8)	2455 (14.4)	< .001	189.75
Thursday	1579 (13.8)	2390 (14.0)	< .001	165.72
Friday	1575 (13.8)	2473 (14.5)	< .001	199.21
Saturday	1678 (14.7)	2268 (13.3)	< .001	88.22
Sunday	1764 (15.4)	2382 (13.9)	< .001	97.71
Weekday	7994 (69.9)	12,427 (72.7)	< .001	**962.32**
Weekend	3442 (30.1)	4670 (27.3)	< .001	185.90
Seasonal patterns *n* (% within group)				
Winter (Dec-Feb)	2845 (24.9)	4428 (25.9)	< .001	**344.55**
Spring (Mars-May)	2860 (25.0)	4284 (25.1)	< .001	283.84
Summer (June-Aug)	3093 (27.0)	4287 (25.1)	< .001	193.18
Autumn (Sep-Nov)	2638 (23.1)	4098 (24.0)	< .001	316.45

One-sample χ^2^ test investigating age group proportion in relation to number of assignments. Percentage in brackets display within group proportion. The χ^2^ values highlighted in bold text are the variables displaying the highest differences between the age groups

The differences were similarly found in a one-sample χ^2^ test of within group variations (not shown in table). Assignments for adults were increasing in the afternoon, being most frequent in the evening between 20:00–23.59 (*p* < 0.001, χ^2^ = 465.32). Assignments within the older adult group were found less than expected in all intervals apart from 08:00–11.59, 12:00–15.59, and 16:00–19.59, the greatest difference found in the forenoon (*p* < 0.001, χ^2^ = 2839.96). Assignments for the older adults were found more than expected during daytime (*p* < 0.001, χ^2^ = 419.16) and in weekdays (*p* < 0.001, χ^2^ = 3519.39), compared to the adults that were found more than expected during nighttime (*p* < 0.001, χ^2^ = 139.27), and in the weekends (*p* < 0.001, χ^2^ = 1811.88). Assignments for older adults were found more than expected in all days of the week, apart from Thursdays, Saturdays and Sundays, and most frequent on Mondays (*p* < 0.001, χ^2^ = 29.17). In contrast, assignments for the adult population were less than expected all week apart from Saturdays and Sundays, most frequently occurring on Sundays (*p* = 0.008, χ^2^ = 17.32). In the seasonal variable, assignments for the older adults were more frequent in the winter, spring, and summer, being most frequent in the winter (*p* = 0.005, χ^2^ = 12.859). Assignments for the adult population were most frequent in the summer (*p* < 0.001, χ^2^ = 36.30).

## Discussion

The aim of the present study was to describe characteristic patterns of dyadic staffed emergency ambulance assignments for older adults aged > 70 years compared with adults aged 18–69 years requiring emergency care at home. The main finding was the preponderance of lower priority levels in the older adult group, in addition to a decreased probability of them receiving the highest priority compared with the adult age group. The reason for this finding might be indicative of the complexity and challenges related to assessing the emergency needs of older adults. Masked symptoms due to polypharmacy, cognitive decline or even natural bodily changes clouds the clinical picture, and make critical conditions hard to detect [20]. In addition, underlying societal tendencies of ageism might be a contributing factor, which are currently being addressed by the World Health Organization to increase the knowledge and competence of health care providers worldwide [1]. Furthermore, the RETTS©, based on vital signs and cutoff points, has been criticized for not being able to differentiate between stable and unstable patients [30]. Swedish EMS RNs report feeling alone in the assessment process, being uncertain about making the right decision, in fear of harming the patient [31]. Another Swedish study similarly concluded that decisions of nonconveyance were difficult and involved a great responsibility [32]. The lack of organizational support, and the risk of being held liable if the wrong decision is made are factors influencing EMS personnel’s decisions [16, 33]. However, a recent review by Yeung et al. [34] found decisions of nonconveyance to be relatively safe for patients. Further attention needs to be paid to why older adults receive lower priorities.

In the present study, 89.4% of the older adults were conveyed to hospital. Conveyance is however not always a suitable option. A major reason for conveyance is the lack of options that results in inadequate transportations to overcrowded emergency departments (EDs) [18, 35]. Frail older adults, being physically intolerant and vulnerable to acute illnesses, do not adapt well to the busy environment at the ED [36]. Moreover, older adults are more likely to have longer waiting times in an ED than younger adults [37, 38]. ED admittance also implies an increased risk for missed diagnoses, delirium, infection [39], pressure ulcers [40], and defaulted care [41–43]. Conveyance of older adults assessed as having low or no acuity might however also be representative of patient participation, the EMS personnel being responsive to the patient’s requests and preferences when deciding about care [17, 44]. In perceived emergency situations, an individual is authorized to call for an ambulance, even for low acuity conditions [45].

Similar to the present case, the problem of a lack of options apart from ED conveyance exists in many countries. A Japanese study [18] suggested focusing on primary care and expanding primary clinic office hours. An Australian study evaluating the establishment of an “acute geriatric outreach service” reported positive results regarding patient safety [46]. In Sweden, older adults expressed satisfaction when offered admittance to a geriatric ward instead of traditional conveyance to EDs when possible, due to previous negative experiences of ED admittance [41]. Shared decision-making was similarly pointed out as beneficial for improving EMS care for older adults in the US [47]. In addition, a possibility for the EMS personnel to consult a physician decreased unnecessary conveyance for low acuity patients in Sweden [48].

In Sweden, mortality is high due to circulatory diseases such as heart failure [11], with the cause of death primarily being related to prior morbidity. The predominance of circulatory clinical assessments in both age groups, provides further support for this finding, implying a future substantial impact on the EMS. Respiratory as well as trauma and nonspecific clinical assessments are reported as being distinctive to older adults worldwide [5, 18, 49, 50]. In the present study, psychiatric assessments were highly associated with the adult age group. This finding is in accordance with Vloet et al. [51], who related behavioral, mental, and neurodevelopmental disorders more frequently to younger nonconveyed patients. However, it can be speculated that psychiatric conditions in older adults become more obscure, meaning that more salient chronic physical conditions are easier to assess. A US study of older adults (aged 55–93 years) found that chronic conditions such as heart or respiratory diseases relate to higher self-reports of depressive symptoms [52]. A recent review focusing on suicidal behavior in older adults concluded that functional disabilities and somatic illness were contributing factors [53].

Concerning the extended on-scene time, research indicates that the clinical complexity of older adults prolongs the overall EMS response time [35, 54]. Harmsen et al. [55] related prolonged on-scene time to more comprehensive care, and additionally suggested a future emphasis on prehospital emergency care, rather than focusing on rapid transportation to EDs. The clinical implications of our findings require further investigation. The actual difference in on-scene time between the youngest and oldest adult groups of patients was around 4.5 min. This finding raises the question whether it is representative of more comprehensive care, or merely a representation of cognitive and functional impairments of older adults prolonging the on-scene time.

The present study found that emergency ambulance assignments for older adults at home were more frequently occurring on Mondays and in the 08:00–11:59 time interval, in accordance with Australian studies [5, 56]. The frequency of assignments on Mondays might be related to home care visits. Fewer visits on the weekends, as well as a greater number of temporary personnel, might prevent the detection of emerging acuity needs. Another reason for this finding may be knowledge about less resources being available on the weekends, making waiting until Monday an option to optimize care. More research is necessary to elucidate the causes underlying this pattern.

### Limitations

Although the relatively large population used for analysis in the present study implies greater statistical power and trustworthiness, some limitations should be noted. The data processed for analysis are dependent on the accuracy of the medical records entered by the EMS RNs. Some relevant data might therefore have been missed due to some inconsistencies in medical records. Because all registry data were anonymized, the possibility of recognizing repeated ambulance transports of the same individual was excluded. It was also not possible to cross-reference community records to identify the number of older adults requiring ambulance care who also utilized home care service, which would have strengthened this study. Another limitation is the lack of causality of the frequent level 2 priorities. This will be further explored in upcoming studies, based on interviews with EMS personnel and older adults.

Since the initial clinical assessment is primarily based on symptoms and vital signs, the assessment might not be consonant with the final ED diagnose. The potential divergence might result in inaccurate clinical data. However, the assessments are anchored in the everyday reality of EMS RNs, and are the results of existing prerequisites, limitations, and competence. Another limitation concerns the evaluation of the logistic regression analysis, and the models’ goodness of fit. In large datasets, the Hosmer-Lemeshow statistics displays deficits. However, the large sample size per se and the robustness of binary logistics might verify the models. Finally, the results of the present study might not be generalizable to other countries, populations, or age groups, although the use of a relatively large sample from the general population allows for broad generalizability to similar contexts.

## Conclusion

Emergency ambulance assignments for older adults at home in a region of Sweden were characterized by a lower priority level preponderance, and a decreased probability for receiving the highest priority. Older adults were more likely to be conveyed, and differentiating initial clinical assessments were circulatory, respiratory, trauma, infection, and nonspecific assessments. Age had an additive effect in relation to on-scene time, and assignments for older adults at home were more frequently occurring during weekdays, on Mondays, and in the 08:00–11:59 interval. Considering the projected increase in the older adult population, the identified patterns may be of use in EMS resource allocation. Health care policy makers need to acknowledge the complex reality of the context, and design adequate and flexible levels of emergency care beneficial to older adults. The issue of the identified priority level divergence warrant further attention in future research, education and clinical practice.

## Data Availability

The datasets used and/or analyzed during the current study are available from the corresponding author on reasonable request.

## Abbreviations

adjOR: Adjusted odds ratio
ALS: Advanced life support
CI: Confidence interval
ED: Emergency department
EMCC: Emergency medical communication center
EMS: Emergency medical services
EMT: Emergency medical technician
OR: Odds ratio
RETTS: Rapid emergency triage and treatment system
RN: Registered nurse
